# Accelerated evolution and spread of multidrug-resistant *Plasmodium falciparum* takes down the latest first-line antimalarial drug in southeast Asia

**DOI:** 10.1016/S1473-3099(19)30394-9

**Published:** 2019-07-22

**Authors:** Didier Ménard, David A Fidock

**Affiliations:** Biology of Host-Parasite Interactions Unit, Malaria Genetics and Resistance Group, Institut Pasteur, INSERM U1201, CNRS ERL9195, Paris, France (DM); and Department of Microbiology and Immunology, and Division of Infectious Diseases, Department of Medicine, Columbia University Irving Medical Center, New York, NY, USA (DAF)

The global fight against malaria has, over the decades, repeatedly been compromised by multidrug-resistant *Plasmodium falciparum* strains that first emerged in southeast Asia.^1^ Successively, these parasites have acquired resistance to chloroquine, sulphadoxine-pyrimethamine, mefloquine, and more recently the artemisinins through point mutations or amplification in genes (*crt*, *dhps*, *dhfr*, *mdr1*, and *kelch13*).^2–6^ Following increased resistance to artesunate plus mefloquine,^7,8^ an early artemisinin-based combined therapy, regional authorities turned increasingly to dihydroartemisinin plus piperaquine. This drug combination was officially adopted in the western provinces of Cambodia in 2008 and the rest of the country in 2010, in Thailand in 2015, and in Vietnam in 2016 (although this artemisinin-based combined therapy was available previously). Warning signs came in 2009 with reports of emerging resistance to artemisinins,^9^ manifesting clinically as delayed rates of parasite clearance^3^ and placing increased selective pressure on the partner drug piperaquine.^10^ In *The Lancet Infectious Diseases*, two studies illustrate the accelerated pace at which resistance of *P falciparum* to dihydroartemisinin plus piperaquine has evolved and spread across southeast Asia, decimating the efficacy of this drug combination.

The first report from Rob van der Pluijm and colleagues^11^ presents interim clinical data from a multicentre, open-label, randomised controlled trial (TRAC2; ) that was done to assess the efficacy, safety, and tolerability of experimental triple artemisinin-based combined therapies compared with two-agent artemisinin-based combined therapies in areas with multidrug-resistant *P falciparum* malaria. Clinical, pharmacological, and genetic data were reported for a cohort of 140 patients with acute *P falciparum* malaria who were treated in 2015–18 with dihydroartemisinin plus piperaquine in sites in Cambodia, Thailand, and Vietnam. PCR-corrected clinical efficacy rates at day 42 were 12.7% in northeastern Thailand, 38.2% in western Cambodia, 73.4% in northeastern Cambodia, and 47.1% in southwestern Vietnam, averaging to a 50.0% treatment failure rate across the region. Treatment failures were far more common than reported a few years ago.^10,12^

Significant increases were also observed across the region in the prevalence of *P falciparum* markers of artemisinin resistance (with the *kelch13* Cys580Tyr [C580Y] variant now at 88%) and piperaquine resistance (*plasmepsin 2* and *plasmepsin 3* amplifications and *crt* mutations, both at 74%), compared with prevalence data from 2011–13 (from the earlier TRAC project). The most striking result was the rapid increase in *crt* mutations, present at a combined prevalence of only 5% in the 2011–13 samples. The risk of treatment failure was strongly associated with the individual *crt* mutations Thr93Ser (T93S), His97Tyr (H97Y), Phe145Ile (F145I), or Ile218Phe (I218F), as well as with *plasmepsin 2* and *plasmepsin 3* amplification. These data, supported by recent clinical, genetic epidemiology, and gene-editing results,^13,14^ provide compelling evidence that these new *crt* mutations can mediate high-grade piperaquine resistance and are driving the increased rates of treatment failure with dihydroartemisinin plus piperaquine.

The second, complementary study by William Hamilton and colleagues^15^ provides detailed insight into the molecular epidemiology of *P falciparum* and the evolution of the artemisinin-resistant and piperaquine-resistant KEL1/PLA1 co-lineage, first identified in samples from 2008 in western Cambodia.^16^ Genome data were analysed from 1673 *P falciparum* clinical samples collected between 2007 and 2018 from patients with malaria in Cambodia, Laos, northeastern Thailand, and Vietnam, combining the TRAC2 and the Genetic Reconnaissance in the Greater Mekong Subregion (GenRe-Mekong) projects. Results showed that KEL1/PLA1 parasites had spread across all the surveyed countries, in several areas exceeding 80% of the local parasite population. Genetic similarity between KEL1/PLA1-type parasites across borders was greater than overall within-country parasite diversity, implying strong selective pressures favouring KEL1/PLA1. Their aggressive expansion across the region was accompanied by the diversification of the KEL1/PLA1 co-lineage into six different subgroups. The three most abundant subgroups carried the mutually exclusive *crt* mutations T93S, H97Y, F145I, or I218F, which had emerged on the *crt* Dd2 haplotype.^15^ This founder haplotype had earlier swept across the region as the primary determinant of chloroquine resistance, and harbours eight point mutations compared with the chloroquine-sensitive wild-type *crt* isoform.^17^ These new variant isoforms were found to co-exist simultaneously in Cambodia, Laos, and Vietnam, suggesting they have a strong selective advantage over the other subgroups (harbouring mostly the piperaquine-sensitive Dd2 isoform). An earlier study reported that the F145I mutation conferred high-level piperaquine resistance in cultured parasites yet had a substantially reduced growth rate in vitro.^14^

Further studies will provide insight into how resistance and fitness contribute to this evolving landscape of soft sweeps that often coalesce around a few isoforms or only one, and will show how quickly this will change as countries move to alternative first-line treatments and whether these new resistance traits expose vulnerabilities in terms of other antimalarial drugs becoming more potent.

In the Greater Mekong Subregion, *P falciparum* parasite populations are highly structured in fragmented forest areas (considered as hotspots of malaria transmission), yet remain interconnected because of intensive human migration and parasite population flow. This epidemiological context, wherein major changes can rapidly occur—for example, following the introduction of new antimalarial drugs, and subsequent extinction or adaptation and recolonisation by the relatively fittest parasite populations—could explain the recent emergence and expansion of new KEL1/PLA1 subgroups flowing across borders. In this regard, findings from these two studies highlight the urgent need to adopt new and effective treatments (such as the triple artemisinin-based combined therapies or the artemisinin-based combined therapy artesunate plus pyronaridine^18^). These findings also demonstrate the advantages of implementing a regional strategy rather than country-specific programmes to address population movements and to integrate regional clinical and genetic surveillance systems into a coordinated campaign, with the goal of achieving malaria elimination in southeast Asia.
